# The Association Between Physical and Mental Health and Face Mask Use During the COVID-19 Pandemic: A Comparison of Two Countries With Different Views and Practices

**DOI:** 10.3389/fpsyt.2020.569981

**Published:** 2020-09-09

**Authors:** Cuiyan Wang, Agata Chudzicka-Czupała, Damian Grabowski, Riyu Pan, Katarzyna Adamus, Xiaoyang Wan, Mateusz Hetnał, Yilin Tan, Agnieszka Olszewska-Guizzo, Linkang Xu, Roger S. McIntyre, Jessica Quek, Roger Ho, Cyrus Ho

**Affiliations:** ^1^ Faculty of Education, Institute of Cognitive Neuroscience, Huaibei Normal University, Huaibei, China; ^2^ Faculty of Psychology, SWPS University of Social Sciences and Humanities, Katowice, Poland; ^3^ Institute of Health Innovation and Technology (iHealthtech), National University of Singapore, Singapore, Singapore; ^4^ Mood Disorders Psychopharmacology Unit, University Health Network, University of Toronto, Toronto, ON, Canada; ^5^ Department of Psychological Medicine, Yong Loo Lin School of Medicine, National University of Singapore, Singapore, Singapore; ^6^ Department of Psychological Medicine, National University Health System, Singapore, Singapore

**Keywords:** anxiety, COVID-19, depression, mask, knowledge, precaution, psychological impact, stress

## Abstract

**Background:**

The physical and mental health of citizens living in a country that encouraged face masks (China) and discouraged face masks (Poland) during the initial stage of the COVID-19 pandemic remained unknown. We conducted a cross-country study to compare the psychological impact of the COVID-19 pandemic on Poles and Chinese. This study aimed to compare the levels of psychological impact of pandemic and levels of anxiety and depression between China and Poland.

**Methods:**

The survey collected information on demographic data, physical symptoms, contact history, and precautionary measures. The psychological impact was assessed using the Impact of Event Scale-Revised (IES-R), and mental health status was assessed by the Depression, Anxiety and Stress Scale (DASS-21). The chi-squared test was used to analyze the differences in categorical variables between the two populations. Linear regression was used to calculate the bivariate associations between independents variables (e.g., physical symptoms and precautionary measures) and dependent variables (e.g., mental health outcomes).

**Results:**

This study included a total of 2,266 respondents from both countries (1,056 Poles and 1,210 Chinese). There were significantly less Polish respondents who wore face masks (Poles: 35.0%; Chinese: 96.8% p < 0.001). Significantly more Polish respondents reported physical symptoms resembling COVID-19 infection (p < 0.001), recent medical consultation (p < 0.01), recent COVID-19 testing (p < 0.001), and hospitalization (p < 0.01). Furthermore, Polish respondents had significantly higher levels of anxiety, depression and stress (p < 0.001) than Chinese. The mean IES-R scores of Poland and China were above the cut-off for post-traumatic stress disorder (PTSD) symptoms. Besides precautionary measures, unemployment, retirement, physical symptoms resembling COVID-19 infection, recent medical consultation or COVID-19 testing, and long daily duration of home confinement were risk factors for PTSD symptoms, anxiety, depression, or stress for Polish respondents.

**Conclusion:**

Use of face masks at the community level may safeguard better physical and mental health during the COVID-19 pandemic. There is a need of health education with scientific information from Polish health authority on the proper use of face masks and reduce social stigma. This study was limited by the respondent sampling method that had compromised the representativeness of samples.

## Introduction

The World Health Organization (WHO) declared the coronavirus disease 2019 (COVID-19) outbreak a pandemic on March 11, 2020 (1). In Asia, China was the first country that was affected by COVID-19. After the outbreak of severe acute respiratory syndrome (SARS), China has built a public health infrastructure to react promptly to the COVID-19 pandemic (2). As of February 2, the number of confirmed cases was 17,205 and the number of death cases was 361 in China (3). Besides enhancing policies on lockdown, social distancing, and personal hygiene (4), China was the first country that enforced compulsory face mask policies for healthy people outside the home environment (5). Due to previous experience with SARS and air pollution (6), wearing a face mask has become a common practice in China and some Asian countries.

Outside China, the number of COVID-19 cases surged in Europe. The local transmission of COVID-19 in Poland was first declared to the WHO on March 10 (7). A new law on specific solutions related to the prevention and eradication of COVID-19 (called “specustawa”, special act) was passed to enforce social distance, prohibition of gathering, closure of business and school, and home confinement (8). As of March 26, the number of confirmed cases of COVID-19 infections was 1221, and there were 16 deaths in Poland (9). In contrast to China, Poland was not affected by SARS in 2003 and Polish medical experts discouraged public from wearing face masks as it was seen as a cultural practice in Asia (10, 11).

Various precautionary measures are adopted during the COVID-19 pandemic. While it is clear that physical distancing can cause loneliness (12), the effects of facemasks has sparked a debate in the medical field and caused confusion. During the initial stage of COVID-19 pandemic, neither the WHO nor Centers for Disease Control and Prevention (CDC) encouraged the usage of face masks for the general public (5, 13). Medical and public health experts from some European countries (e.g., United Kingdom) believed there was no direct evidence of airborne transmission of COVID-19 (14). In contrast, respiratory clinicians and public health experts in China argued that lack of evidence does not equate to evidence of ineffectiveness of face masks (15). The use of face masks, by most people in Hong Kong have played an important role in controlling the spread of COVID-19 (16). Although air quality and ventilation experts believed wearing face mask could offer psychological benefits (14), the mixed opinion, contradictory messaging, and shortage of face masks could possibly lead to public anxiety and confusion. Furthermore, the public might be concerned about emerging clinical reports about the spread of COVID-19 by asymptomatic carriers (17).

One of the methods to study the possible association between wearing face masks and mental health parameters during the COVID-19 pandemic is to compare mental health of two countries with different views and practices with similar mental health status before the COVID-19 pandemic (18). Recent studies reported the mental health of Chinese during the pandemic (19–21) but there was no similar study on Poles. We hypothesized that (a) More Chinese respondents would prefer to wear face masks as a preventive measure; (b) the frequencies of physical symptoms and levels of psychological impact, depression, anxiety, and stress were different between Polish and Chinese respondents; (c) different factors were associated with psychological impact, depression, anxiety, and stress in Polish and Chinese respondents.

## Methods

### Study Design and Study Population

We conducted a cross-country study to compare the psychological impact of the COVID-19 pandemic on Poles and Chinese. The study was conducted from January 31 to February 2 in China and March 22 to March 26 in Poland, during the initial stages of the epidemic in both countries. A respondent driven sampling strategy focused on recruiting the general public during the COVID-19 pandemic was utilized. The formula to calculate sample size is listed as follows (22):

The sample size *n* and margin of error *E* are given by

x=Z(c100)2r(100−r)n=Nx((N−1)E2+x)E=Sqrt[(N−n)xn(N−1)]

where N is the population size, r is the fraction of responses, and Z (c/100) is the critical value for the confidence level c. The minimum sample size for China was 664 (margin error is 5%, confidence level at 99%; the population of China is 1,439,525,218; response distribution: 50%) (22). The minimum sample size for Poland was 384 (margin error is 5%, confidence level at 99%; the population of Poland is 37,845,009) (22). Inclusion criteria for respondents were access to the Internet, able to read Chinese or Polish, residing in China or Poland during the recruitment period. Exclusion criteria were no access to the Internet, illiteracy, and not residing in China or Poland at the time of recruitment.

### Procedure

In order to comply with social distancing and lockdown measures in both countries, potential respondents were electronically invited by existing study respondents by the respondent sampling technique. They completed the questionnaires through an online survey platform (Google Forms Online Survey in Poland and “SurveyStar”, Changsha Ranxing Science and Technology, Shanghai in China). The Institutional Review Board of the SWPS University (Poland) (IRB Reference Number WKEB62/04/2020), Huaibei Normal University (China) (HBU-IRB-2020-002) approved the research proposals. All respondents provided informed consent and anonymous data were kept confidential.

### Outcomes

This study used the National University of Singapore COVID-19 questionnaire, and its psychometric properties had been established during the COVID-19 outbreak (23) and pandemic (24). The National University of Singapore COVID-19 questionnaire consisted of questions that covered five main areas: (1) demographic data; (2) physical symptoms related to COVID-19 in the past 14 days; (3) contact history with COVID-19 in the past 14 days; (4) knowledge and concerns about COVID-19; and (5) precautionary measures against COVID-19 in the past 14 days.

Demographic data about age, gender, education, household size, and marital status were collected. Physical symptoms related to COVID-19 included breathing difficulty, chills, coryza, cough, dizziness, fever, headache, myalgia, sore throat, and fever. Respondents also rated their physical health status and stated their history of chronic medical illness. Health service utilization variables in the past 14 days included consultation with a doctor in the clinic, quarantine experience, and recent testing for COVID-19. Precautionary measures against COVID-19 included covering mouth when coughing and sneezing, hand hygiene, and wearing a face mask regardless of the presence or absence of symptoms. The respondents were asked the average number of hours of home confinement per day during the COVID-19 pandemic.

The psychological impact of COVID-19 was measured using the Impact of Event Scale-Revised (IES-R). The IES-R was validated in Polish and Chinese population for determining the extent of psychological impact after exposure to a recent event that might threaten survival (i.e., the COVID-19 pandemic) (25–27). This 22-item questionnaire which is composed of three subscales, aims to measure the mean avoidance, intrusion, and hyperarousal (28). The total IES-R score is divided into 0–23 (normal), 24–32 (mild psychological impact), 33–36 (moderate psychological impact) and >37 (severe psychological impact) (29). The total IES-R score > 24 suggests the presence of post-traumatic stress disorder (PTSD) symptoms (30). The Chinese version of IES-R was a valid and reliable measure of psychological distress. Based on factor analysis and subscale correlation, the three subscales of the Chinese version of IES-R were highly related and reliability was verified (31). The Chinese version of IES-R demonstrated external validity with significant correlation with General Health Questionnaire (31). Similarly, the Polish version of IES-R was found to be reliable and valid method. The factor structure is similar to the proposed theoretical structure (32). Principal component analysis identified three factors including intrusion, hyperarousal, and avoidance (32). IES-R was previously used in research related to the COVID-19 epidemic (23, 33, 34). We also assessed the reliability and validity of IES-R for this study. For reliability, the internal consistency or homogeneity of items of IES-R was measured by the Cronbach’s alpha. Cronbach’s alpha of 0.70 or higher is considered “acceptable” in most social science research (35). In this study, the Cronbach’s alpha for Chinese version of IES-R was 0.949 and Cronbach’s alpha for Polish version of IES-R was 0.883. The Spearman-Brown split half reliability coefficient is used to estimate full test reliability based on split-half reliability measures and the Spearman-Brown coefficient of 0.80 or higher is considered to demonstrate good reliability. In this study, the Spearman-Brown coefficient for Chinese version of IES-R was 0.916 and Cronbach’s alpha for Polish version of IES-R was 0.87. For face validity, there was 100% completion rate for both Chinese and Polish respondents and indicated good comprehensibility and interpretability of IES-R. Construct validity was assessed by the confirmatory factor analysis (CFA). The goodness-of-fit indices revealed a good fit of the data model [Chinese version of IES-R: χ2/d.f. = 2.467 (<3: excellent), RMSEA = 0.07 (<0.1: acceptable), CFI = 0.937 (>0.9: acceptable), IFI = 0.937 (>0.9 acceptable), NFI = 0.899 (>0.90: acceptable); Polish version of IES-R: χ2/d.f. = 2.269 (<3: excellent), RMSEA = 0.065 (<0.1: acceptable), CFI = 0.917 (>0.9: acceptable), IFI = 0.919 (>0.9 acceptable), NFI = 0.863 (>0.90: acceptable)].

The mental health status of respondents was measured using the Depression, Anxiety and Stress Scale (DASS-21) and calculation of scores was based on a previous study (36). The DASS-21 was recommended to meaningfully compare the relationships between variables across different ethnic groups (37). DASS-21 has been demonstrated to be a reliable and valid measure in assessing mental health in Poles (37) and Chinese (38–40). Previous study reported that each of the three subscales of DASS-21 demonstrated good internal consistency, test-retest reliability and convergent validity with other established scales such as Chinese version of Beck Depression Inventory (BDI) and State-Trait Anxiety Inventory (STAI) (40). Confirmatory factor analysis of the assumed three-factor model of Polish version of DASS-21 suggested that the model was appropriate for Poles (37). DASS-21 was previously used in research related to the COVID-19 epidemic (23, 33, 34). We also assessed the reliability and validity of DASS-21 for this study. For internal consistency, the Cronbach’s alpha of Chinese version of DASS-21 was listed as follows: DASS-21 stress: 0.888, DASS-21 anxiety: 0.845, DASS-21 depression: 0.878. The Cronbach’s alpha of Polish version of DASS-21 was listed as follows: DASS-21 stress: 0.890, DASS-21 anxiety: 0.854, DASS-21 depression: 0.886. For split-half reliability, the Spearman-Brown coefficient for Chinese version of DASS-21 was 0.929 and Cronbach’s alpha for Polish version of DASS-21 was 0.937. For face validity, there was 100% completion rate for both Chinese and Polish respondents and indicated good comprehensibility and interpretability of DASS-21. For construct validity, the CFA revealed a good fit of the data model [Chinese version of DASS-21: χ2/d.f. = 2.382 (< 3: excellent), RMSEA = 0.068 (<0.1: acceptable) CFI = 0.944 (>0.9: acceptable), IFI = 0.944 (>0.9 acceptable), NFI = 0.908 (>0.90: acceptable); Polish version of DASS-21: χ2/d.f. = 2.201 (<3: excellent), RMSEA = 0.063 (<0.1: acceptable), CFI = 0.950 (>0.9: acceptable), IFI = 0.951 (>0.9 acceptable), NFI = 0.913 (>0.90: acceptable)].

### Statistical Analysis

Descriptive statistics were calculated for demographic characteristics, physical symptom, and health service utilization variables, contact history variables, and precautionary measure variables. The scores of IES-R and DASS subscales were expressed as mean and standard deviation. To analyze the differences in psychological impact, levels of depression, anxiety and stress, the independent sample t-test was used to compare the mean score between the Polish and Chinese respondents. Percentages of response to other questions were calculated according to the number of respondents per response to the number of total responses of a question and presented as categorical variables. The chi-squared test was used to analyze the differences in categorical variables between the two samples. We used linear regressions to calculate the bivariate associations between independent variables including demographic characteristics, physical symptoms and health status, and precautionary measures, and dependent variables including the IES-S score and DASS stress, anxiety, and depression subscale scores for the Poles and Chinese separately. The maximum probability of a Type I error remains alpha (p < 0.05). In this study, there are three levels of p-values: p < 0.05, p < 0.01, and p < 0.001 for the significant regression analysis results. For example, a *p*-value of 0.01 would mean there is a 1% chance of committing a Type I error [2]. Statistical analysis was performed on SPSS Statistic 21.0.

## Results

### Comparison Between the Polish and Chinese Respondents and Their Mental Health Status

For the study in Poland, we received responses from 1,064 respondents, and 8 respondents did not complete the questionnaires. Eventually, we included 1,056 respondents from Poland who had completed the questionnaires (99.2%). For the China sample, we excluded 94 incomplete questionnaires, which yielded 1,210 of a total of 1,304 (92.79%) valid questionnaires from China. As a result, there were a total of 2,266 individual respondents who participated in both countries.

**Figure 1:**
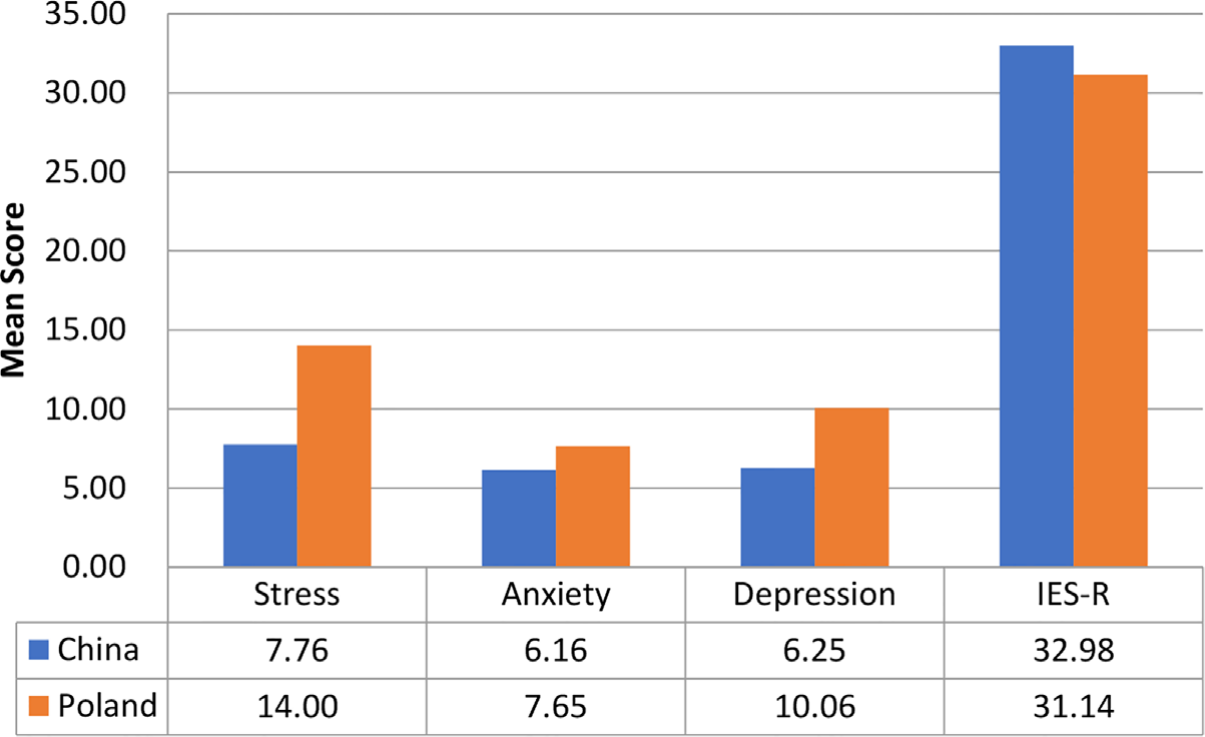
Comparison of the mean scores of DASS-stress, anxiety, and depression subscales as well as IES-R scores between Polish and Chinese respondents (N = 2,266).

**Figure 1** compares the mean scores of DASS-stress, anxiety, and depression subscales and IES-R scores between the Polish and Chinese respondents. For the DASS-stress subscale (M_China_ = 7.76, SD_China_ = 7.74; M_Poland_ = 14.00, SD_Poland_ = 10.09), Chinese had significantly lower stress scores (t = 16.32, *p* < 0.001, 95%CI 5.50 to 7.00). For the DASS-anxiety subscale (M_China_ = 6.16, SD_China_ = 6.57; M_Poland_ = 7.65, SD_Poland_ = 8.12), Chinese had significantly lower anxiety scores (t = 4.76, *p* < 0.001, 95% CI 0.87 to 2.11). For the DASS-depression subscale (M_China_ = 6.25, SD_China_ = 7.16; M_Poland_ = 10.06, SD_Poland_ = 9.23), Chinese had significantly lower depression scores (t = 10.88, *p* < 0.001, 95% CI 3.13 to 4.51). For IES-R (M_China_ =32.98, SD_China_ = 15.42; M_Poland_ = 31.14, SD_Poland_ = 13.59), Chinese had significantly higher IES-R scores (t = −3.03, p < 0.01, 95% CI −3.04 to −0.65). Nevertheless, the mean IES-R scores of both countries were higher than 24 points, indicating the presence of PTSD symptoms in Polish and Chinese respondents.

### Demographic Characteristics and Its Association With Psychological Impact and Adverse Mental Health Status

The majority of Polish respondents were women (76.1%), of middle adulthood with the average age of 31 to 40 years (45.4%), married (55.5%) having a household size of 3–5 people (57.4%), employed (84.5%), and well educated (72.9% with a bachelor or higher degree). Similarly, the majority of Chinese respondents were women (67.3%), married (76.4%), of young adulthood with age 22 to 30 years (53.2%), having a household size of 3–5 people (80.7%), students (52.8%), and well educated (87.9 with a bachelor or higher degree) (see **Table 1**).

**Table 1:** Comparison of demographic characteristics between Polish and Chinese respondents (N = 2,266).

Demographic characteristics	Poland (N = 1,056)		China (N = 1,210)
	N (%)		N (%)
**Gender**			
Male	252 (23.9)		396 (32.7)
Female	804 (76.1)		814 (67.3)
**Age range**			
[12–21]	74 (7.0)		344 (28.4)
[22–30]	217 (20.5)		643 (53.2)
[31–40]	479 (45.4)		94 (7.8)
[41–49]	216 (20.5)		90 (7.4)
>50	70 (6.6)		39 (3.2)
**Household size**			
Six people or more	50 (4.7)		171 (14.1)
Three to five people	606 (57.4)		976 (80.7)
Two people	265 (25.1)		52 (4.3)
One person	135 (12.8)		11 (0.9)
**Education level**			
Primary school and below	5 (0.5)		10 (0.8)
Junior high school	32 (3.0)		55 (4.6)
High school	249 (23.6)		81 (6.7)
University (Bachelor, Master, Doctorate)	770 (72.9)		1,064 (87.9)
**Employment status**			
Student	88 (8.3)		639 (52.8)
Unemployed	47 (4.5)		67 (5.5)
Farming	0 (0)		24 (2.0)
Retired	28 (2.7)		7 (0.6)
Employed	893 (84.5)		473 (39.1)
**Marital status**			
Married	586 (55.5)		925 (76.4)
Single	460 (43.6)		273 (22.6)
Divorced/Separated	0 (0)		9 (0.7)
Widowed	10 (0.9)		3 (0.3)

For Polish respondents, the male gender was significantly associated with the lower score of IES-R and DASS-21 subscale scores (p < 0.01) (see **Table 2**). In contrast, male gender was significantly associated with the lower score of IES-R but higher DASS-21 subscale scores in Chinese respondents (p < 0.05). Notwithstanding, there were other differences between Polish and Chinese respondents. Unemployment and retirement were significantly associated with higher IES-R scores in Poles (p < 0.05). Higher education levels were associated with lower DASS-21 depression scores (p < 0.01) in Poles but higher IES-R scores in Chinese (p < 0.05). Polish students were significantly associated with higher DASS-21 depression scores (p < 0.001) while Chinese students were significantly associated with higher IES-R, DASS-21 stress and anxiety scores (p < 0.05).

**Table 2:** Association between demographic variables and the psychological impact of the 2019 coronavirus disease (COVID-19) outbreak as well as adverse mental health status between Polish and Chinese respondents during the epidemic (N = 2,266).

Demographic variables	Poland								China							
	Impact of event		Stress		Anxiety		Depression		Impact of event		Stress		Anxiety		Depression	
	*B*	*T*	*B*	*t*	*B*	*t*	*B*	*T*	*B*	*t*	*B*	*t*	*B*	*t*	*B*	*t*
**Gender**																
Male	−0.54	−6.23***	−0.50	−6.23***	−0.58	−5.77***	−0.31	−3.37**	−0.20	−2.56*	0.10	2.33*	0.19	2.64**	0.12	2.13*
Female	Reference		Reference		Reference		Reference		Reference		Reference		Reference		Reference	
**Age (years)**																
12–21.4	−0.35	−1.72	0.08	0.42	0.23	1.00	0.51	2.42*	0.21	1.00	0.08	0.65	0.10	0.51	0.06	0.39
21.4–30.8	−0.40	−2.39*	−0.02	−0.10	−0.21	−1.09	−0.04	−0.22	0.09	0.45	0.12	1.01	0.07	0.36	0.18	1.15
30.8–40.2	−0.25	−1.59	−0.02	−0.16	−0.18	−1.02	−0.20	−1.22	−0.17	−0.73	−0.07	−0.52	−0.16	−0.72	−0.06	−0.36
40.2–49.6	−0.26	−1.53	−0.10	−0.61	−0.27	−1.42	−0.21	−1.18	−0.16	−0.69	−0.12	−0.82	−0.23	−1.05	−0.16	−0.89
>49.6	Reference		Reference		Reference		Reference		Reference		Reference		Reference		Reference	
**Marital status**																
Single	−0.07	−0.19	−0.002	−0.01	0.29	0.65	−0.16	−0.40	−0.12	−0.33	0.02	0.10	0.38	1.11	0.12	0.44
Married	0.03	0.07	0.03	0.09	0.35	0.78	−0.32	−0.78	0.002	0.01	0.12	0.58	0.47	1.40	0.22	0.82
Widowed	Reference		Reference		Reference		Reference		Reference		Reference		Reference		Reference	
**Household size**																
Six people or more	0.06	0.31	0.08	0.41	0.29	1.23	0.08	0.38	0.38	0.97	−0.23	−0.99	−0.17	−0.46	−0.19	−0.67
Three to five people	0.12	1.03	0.06	0.59	0.16	1.17	0.10	0.83	0.25	0.65	−0.20	−0.88	−0.12	−0.35	−0.09	−0.31
Two people	0.14	1.04	0.05	0.44	0.18	1.18	0.13	0.96	0.41	0.99	−0.33	−1.35	−0.18	−0.46	−0.21	−0.69
One person	Reference		Reference		Reference		Reference		Reference		Reference		Reference		Reference	
**Employment status**																
Unemployed	0.51	2.79**	0.31	1.85	0.26	1.22	0.27	1.45	0.13	0.81	0.12	1.26	0.21	1.38	0.16	1.33
Retired	0.59	2.51*	−0.06	−0.29	0.13	0.48	0.16	0.66	−0.76	−1.60	−0.37	−1.34	−0.55	−1.24	−0.48	−1.36
Student	−0.04	−0.29	0.17	1.35	0.33	2.10*	0.74	5.23***	0.20	2.71**	0.11	2.40*	0.16	2.25*	0.08	1.46
Employed	Reference		Reference		Reference		Reference		Reference		Reference		Reference		Reference	
**Education level**																
Junior high school	−0.01	−0.02	−0.62	−1.14	−0.96	−1.42	−1.71	−2.79**	1.08	2.53*	0.14	0.54	0.26	0.64	0.10	0.31
High school	0.35	0.63	−0.37	−0.72	−0.59	−0.94	−1.70	−2.97**	0.88	2.11*	0.10	0.39	0.24	0.62	0.03	0.10
University (Bachelor, Master, Doctorate)	0.28	0.50	−0.38	−0.76	−0.84	−1.34	−1.91	−3.35**	1.04	2.64**	0.14	0.58	0.17	0.47	0.03	0.09
Primary school and below	Reference		Reference		Reference		Reference		Reference		Reference		Reference		Reference	

*P < 0.05; **p < 0.01; ***p < 0.001.

### Physical Symptoms, Health Status, and Its Association With Psychological Impact and Adverse Mental Health Status

For physical symptoms resembling COVID-19 and health status, there were a significantly higher proportion of Polish respondents who reported fever (p < 0.001), breathing difficulty (p < 0.001), coryza (p < 0.001), sore throat (p < 0.001), recent consultation with a doctor (p < 0.01), recent COVID-19 testing (p < 0.001), recent hospitalization (p < 0.01), chronic illness (p < 0.01), indirect contact with a confirmed case of COVID-19 (p < 0.05), and contact with infected materials (p < 0.001) as compared to Chinese (see **Table 3**). Significantly less Chinese respondents reported good health status (p < 0.001) and more Chinese respondents had recent quarantine experience (p < 0.01). Of the Polish respondents, 614 respondents (58.1%) had no symptom, 227 respondents (21.5%) had one symptom, 126 respondents (11.9%) had two symptoms, and 52 (4.9%) respondents had three symptoms. Of the Chinese respondents, 793 respondents reported no symptoms (60.81%), 182 respondents reported one symptom (15.04%), 114 respondents reported two symptoms (9.42%), and 68 respondents reported three symptoms (5.62%).

**Table 3:** Comparison of physical health status in the past 14 days of the 2019 coronavirus disease (COVID-19) outbreak between Polish and Chinese respondents during the epidemic (N = 2,266).

Variable	Poland (N = 1,056)	China (N = 1,210)	Chi-square (χ^2^)	p-value
	N(%)	N(%)		
**Persistent fever (>38°C for at least one day)**				
Yes	39 (3.7)	6 (0.5)	29.615	p < 0.001***
No	1,017 (96.3)	1,204 (99.5)		
**Chills**				
Yes	30 (2.8)	42 (3.5)	0.728	p = 0.394
No	1,026 (97.2)	1,168 (96.5)		
**Myalgia**				
Yes	71 (6.7)	95 (7.9)	1.056	p = 0.304
No	985 (93.3)	1,115 (92.1)		
**Cough**				
Yes	163 (15.4)	168 (13.9)	1.088	p = 0.297
No	893 (84.6)	1,042 (86.1)		
**Breathing difficulty**				
Yes	35 (3.3)	5 (0.4)	27.370	p < 0.001***
No	1,021 (96.7)	1,205 (99.6)		
**Coryza**				
Yes	265 (25.1)	205 (16.9)	22.798	p < 0.001***
No	791 (74.9)	1,005 (83.1)		
**Sore throat**				
Yes	203 (19.2)	139 (11.5)	26.333	p < 0.001***
No	853 (80.8)	1,071 (88.5)		
**Consultation with a doctor in the last 14 days**				
Yes	243 (23.0)	42 (3.5)	195.813	p < 0.001***
No	813 (77.0)	1,168 (96.5)		
**Current self-rating of health status**				
Poor or very poor	11 (1.0)	11 (1.0)	150.042	p < 0.001***
Average	103 (9.8)	372 (30.7)		
Good or very good	942 (89.2)	827 (68.3)		
**Chronic illness**				
Yes	223 (21.1)	78 (6.4)	105.368	p < 0.001***
No	833 (78.9)	1,132 (93.6)		
**Recent testing for COVID-19 in the last 14 days**				
Yes	243 (23.0)	11 (0.9)	276.772	p < 0.001***
No	813 (77.0)	1,199 (99.1)		
**Recent hospitalization in the past 14 days**				
Yes	17 (1.6)	4 (0.3)	10.051	p < 0.01**
No	1039 (98.4)	1,206 (99.7)		
**Recent quarantine in the last 14 days**				
Yes	6 (0.6)	26 (2.1)	10.118	p < 0.01**
No	1,050 (99.4)	1,184 (97.9)		
**Close contact with an individual with confirmed infection with COVID-19**				
Yes	6 (0.6)	4 (0.3)	NA	NA
No	1,050 (99.4)	1,206 (99.7)		
**Indirect contact with an individual with confirmed infection with COVID-19**				
Yes	14 (1.3)	6 (0.5)	4.439	p = 0.035*
No	1,042 (98.7)	1,204 (99.5)		
**Contact with infected material by COVID-19**				
Yes	164 (15.5)	12 (1.0)	166.377	p < 0.001***
No	892 (84.5)	1,198 (99.0)		
**Concerns about other family members contracting COVID-19**				
Yes	893 (84.6)	909 (75.2)		
No	163(15.4)	291 (24.0)	NA	NA
No other family members	0 (0)	10 (0.8)		

*p < 0.05, **p <0.01, ** p <0.001.

For both countries, linear regression showed that chills and myalgia were significantly associated with higher IES-R scores, DASS-21 stress, anxiety, and depression subscale scores after adjustment for age, gender, and education levels (p < 0.05) (see **Table 4**). Cough and poor physical status were significantly associated with higher DASS-21 stress, anxiety, and depression subscale scores (p < 0.05). The presence of chronic illness and sore throat were significantly associated with higher IES-R scores, stress, and anxiety (p < 0.05). Breathing difficulty was associated with higher DASS-21 anxiety and depression scores (p < 0.01). Contact with infected material by COVID-19 was associated with higher DASS-21 anxiety scores (p < 0.05). Concerns about other family members contracting COVID-19 were significantly associated with higher IES-R scores (p < 0.05). Recent quarantine was not associated with IES-R and DASS-21 subscale score (p > 0.05). There were other differences between the two countries. Recent consultation with a doctor and COVID-19 testing was significantly associated with higher DASS-21 anxiety scores in Poles only (p < 0.01). Close contact with confirmed case of COVID-19 infection was associated with depression in Chinese only (p < 0.05).

**Table 4:** Association between physical health status in the past 14 days and contact history, and the psychological impact of 2019 coronavirus disease outbreak (COVID-19) as well as adverse mental health status between Polish and Chinese respondents during the epidemic with adjustment for age, gender, and education levels (N = 2266).

Variables	Poland																	China							
	Impact of Event			Stress				Anxiety					Depression					Impact of Event		Stress		Anxiety		Depression	
	*B*	*t*		*B*		*T*		*B*		*t*			*B*			*t*		*B*	*t*	*B*	*T*	*B*	*t*	*B*	*t*
**Persistent Fever (>38°C for at least one day)**																									
Yes	0.27	1.35		0.12		0.64		0.27		1.19			0.05			0.23		−0.23	−0.44	0.40	1.34	1.23	2.60*	0.98	2.57*
No	Reference			Reference				Reference					Reference					Reference		Reference		Reference		Reference	
**Chills**																									
Yes	0.63	2.80**		0.81		3.90***		0.63		2.44*			0.53			2.23*		0.46	2.34*	0.44	3.84***	0.60	3.31**	0.41	2.84**
No	Reference			Reference				Reference					Reference					Reference		Reference		Reference		Reference	
**Myalgia**																									
Yes	0.31	2.07*		0.56		4.06***		0.49		2.86**			0.52			3.31**		0.63	4.77***	0.43	5.60***	0.69	5.61***	0.50	5.08***
No	Reference			Reference				Reference					Reference					Reference		Reference		Reference		Reference	
**Cough**																									
Yes	0.20	1.96		0.31		3.22**		0.48		4.02***			0.39			3.59***		0.33	3.23**	0.19	3.11**	0.29	2.97**	0.21	2.70**
No	Reference			Reference				Reference					Reference					Reference		Reference		Reference		Reference	
**Breathing difficulty**																									
Yes	0.48	2.26*		0.60		3.14**		1.01		4.24***			0.82			3.74***		0.88	1.58	0.57	1.74	1.63	3.15**	1.28	3.08**
No	Reference			Reference				Reference					Reference					Reference		Reference		Reference		Reference	
**Coryza**																									
Yes	0.02	0.28		0.12		1.44		0.16		1.60			0.09			0.96		0.39	4.11***	0.25	4.46***	0.46	5.18***	0.33	4.70***
No	Reference			Reference				Reference					Reference					Reference		Reference		Reference		Reference	
**Sore throat**																									
Yes	0.21	2.22*		0.25		2.84**		0.38		3.52***			0.19			1.94		0.34	2.99**	0.16	2.45*	0.35	3.35**	0.17	2.08*
No	Reference			Reference				Reference					Reference					Reference		Reference		Reference		Reference	
**Consultation with doctor in the clinic in the last 14 days**																									
Yes	0.14	1.58		0.11		1.37		0.27		2.61**			0.16			1.68		−0.06	−0.31	0.17	1.47	0.38	2.08*	0.22	1.48
No	Reference			Reference				Reference					Reference					Reference		Reference		Reference		Reference	
**Recent hospitalization in the past 14 days**																									
Yes	0.05	0.15		−0.08		−0.28		0.28		0.81			0.47			1.49		0.78	1.25	0.32	0.87	1.23	2.12*	−0.28	−0.60
No	Reference			Reference				Reference					Reference					Reference		Reference		Reference		Reference	
**Recent testing for COVID-19 in the past 14 days**																									
Yes	0.14	1.58		0.11		1.37		0.27		2.61**			0.16			1.68		−0.18	−0.48	−0.07	−0.31	0.22	0.64	0.02	0.06
No	Reference			Reference				Reference					Reference					Reference		Reference		Reference		Reference	
**Recent quarantine in the past 14 days**																									
Yes	−0.15	−0.30		−0.18		−0.40		−0.36		−0.62			−0.12			−0.23		0.32	1.30	−0.01	−0.06	0.03	0.13	−0.11	−0.59
No	Reference			Reference				Reference					Reference					Reference		Reference		Reference		Reference	
**Current self-rating of health status**																									
Very poor	0.75	2.04*		0.86		2.56*		1.16		2.76**			1.58			4.16***		1.39	1.13	3.63	5.03***	3.35	2.94**	3.56	3.88***
Poor																		1.69	1.77	0.13	0.57	0.65	1.81	0.36	1.23
Average	0.50	3.92***		0.56		4.76***		0.75		5.26***			0.66			5.08***		0.37	4.73***	0.19	4.28***	0.41	5.70***	0.26	4.63***
Good or very good	Reference			Reference				Reference					Reference					Reference		Reference		Reference		Reference	
**Chronic illness**																									
Yes	0.27	2.91**		0.22		2.53*		0.35		3.32**			0.08			0.85		0.30	2.02*	0.24	2.77**	0.48	3.58***	0.38	3.51***
No	Reference			Reference				Reference					Reference					Reference		Reference		Reference		Reference	
**Close contact with an individual with confirmed infection with COVID-19**																									
Yes	0.35	0.70		0.15		0.33		−0.02			−0.04		−0.29		−0.55			0.53 0.84		0.32 0.87		0.98 1.68		0.97 2.10*	
No	Reference			Reference				Reference					Reference					Reference		Reference		Reference		Reference	
**Indirect contact with an individual with confirmed infection with COVID-19**																									
Yes	−0.35	−1.05		−0.38		−1.25		−0.24			−0.63		−0.39		−1.13			−0.06 −0.11		-0.27 -0.89		-0.28 -0.59		-0.37 -0.96	
No	Reference			Reference				Reference					Reference					Reference		Reference		Reference		Reference	
**Contact with infected material by COVID-19**																									
Yes	0.17	1.64			0.21		2.15*		0.24			2.04*		0.17			1.57	0.36 1.00		0.41 1.91		0.98 2.93**		0.81 3.02**	
No	Reference			Reference				Reference					Reference					Reference		Reference		Reference		Reference	
**Concerns about other family members contracting COVID-19**																									
Yes	0.27		2.57*		0.17		1.76		0.23			1.91		0.10			0.90	0.34 4.15***		0.06 1.19		0.10 1.28		0.04 0.68	
No	Reference			Reference				Reference					Reference					Reference		Reference		Reference		Reference	

*p<0.05, **p<0.01, ***p<0.001.

### Precautionary Measures About COVID-19 and Its Association With Psychological Impact and Adverse Mental Health Status

Polish and Chinese respondents demonstrated significantly different precautionary measures (see **Table 5**). There were significantly more Chinese respondents who would cover their mouths when coughing and sneezing (p < 0.001), practice hand hygiene (p < 0.05), wear masks (p < 0.001), spent most of the time at home (20–24 h) (p < 0.001), and satisfied with health information (p < 0.001) than Poles.

**Table 5:** Comparison of precautionary measures in the past 14 days of the 2019 coronavirus disease (COVID-19) outbreak between Polish and Chinese respondents during the epidemic (N = 2,266).

Variables	Poland (N = 1,056)		China (N = 1,210)	Chi-square (χ^2^)	p-value	
	N (%)		N (%)			
**Covering mouth when coughing or sneezing**					
Yes	786 (74.4)		1,159 (95.8)	211.453	p < 0.001***	
No	270 (25.6)		51 (4.2)			
**Washing hands with soap and water**					
Yes	1,009 (95.5)		1,177 (97.3)	4.918	p = 0.027*	
No	47 (4.5)		33 (2.7)			
**Wearing mask or protective gloves regardless of presence or absence of symptoms**					
Yes	370 (35.0)		1,171 (96.8)	987.842	p < 0.001***	
No	686 (65.0)		39 (3.2)			
**Average number of hours staying at home per day to avoid COVID-19**					
[0–9]	90 (8.5)		29 (2.4)	230.918	p < 0.001***	
[10–19]	277 (26.2)		77 (6.4)			
[20–24]	689 (65.3)		1,104 (91.2)			
**Satisfaction with the amount of health information available about COVID-19**						
Satisfied	466 (44.1)		908 (75.0)			
Not satisfied	200 (19.0)		251 (20.8)	399.926	p < 0.001***	
Do not know	390 (36.9)		51 (4.2)			

*p < 0.05, ***p < 0.001.

Linear regression analysis showed that hand hygiene practice was associated with lower DASS-21 anxiety scores in Polish and Chinese respondents after adjustment for age, gender, and education levels (p < 0.05) (see **Table 6**). Use of face mask and satisfaction with health information were not associated with mental health parameters in Poles and Chinese (p > 0.05). For Poles, staying at home for 10–19 h was significantly associated with lower IES-R and DASS-21 stress scores (p < 0.05) as compared to home confinement for 20–24 h. This association was not found in Chinese, suggesting that the daily duration of home quarantine might not have an adverse effect on mental health in Chinese.

**Table 6:** Comparison between associations of precautionary measures in the past 14 days, health information and the psychological impact of the 2019 coronavirus disease (COVID-19) outbreak, as well as adverse mental health status between Polish and Chinese respondents during the epidemic with adjustment for age, gender, and education (N = 2,266).

Variables	Poland													China											
	Impact of Event			Stress			Anxiety				Depression			Impact of Event			Stress			Anxiety			Depression		
	*B*	*T*		*B*	*T*		*B*	*t*			*B*	*t*		*B*		*t*	*B*	*t*		*B*	*t*		*B*	*t*	
**Covering mouth when coughing or sneezing**																									
Yes	−0.01	−0.14		−0.11	−1.43		−0.04	−0.37			−0.17	−1.91		0.10		0.57	0.04	0.40		−0.15	−0.90		−0.06	−0.46	
No	Reference			Reference			Reference				Reference			Reference			Reference			Reference			Reference		
**Washing hands with soap and water**																									
Yes	−0.27	−1.49		−0.32	−1.89		−0.49	−2.35*			−0.33	−1.74		−0.25		−1.15	−0.30	−2.35*		−0.45	−2.19*		−0.33	−2.00*	
No	Reference			Reference			Reference				Reference			Reference			Reference			Reference			Reference		
**Wearing mask or protective gloves regardless of presence or absence of symptoms**																									
Yes	0.12	1.41		0.09	1.22		0.02	0.18			−0.03	−0.32		−0.05		−0.23	−0.16	−1.36		−0.36	−1.92		−0.29	−1.92	
No	Reference			Reference			Reference				Reference			Reference			Reference			Reference			Reference		
**Average number of hours staying at home per day to avoid COVID-19**																									
0–9 h	0.04	0.28		0.04	0.29		−0.15	−0.98			0.01	0.04		−0.35		−1.49	−0.27	−1.94		−0.41	−1.87		−0.30	−1.71	
10–19 h	−0.23	−2.60*		−0.23	−2.93**		−0.10	−1.01			−0.13	−1.50		0.09		0.62	−0.01	−0.12		0.05	0.34		−0.03	−0.30	
20–24 h	Reference			Reference			Reference				Reference			Reference			Reference			Reference			Reference		
**Satisfaction with the amount of health information available about COVID-19**
Satisfied	−0.10		−1.13	−0.02		−0.20	−0.07		−0.72	0.03			0.36	0.12	0.65		−0.03		−0.31	−0.12		−0.69	−0.06		−0.47
Not satisfied	−0.09		−0.88	−0.02		−0.16	−0.22		−1.78	0.07			0.67	0.37	1.92		0.12		1.09	0.11		0.59	0.13		0.93
Do not know	Reference			Reference			Reference			Reference				Reference			Reference			Reference			Reference		

## Discussion

To our best knowledge, this is the first study that compared the psychological impact and mental health Poland and China that adopted very different precautionary measures during the COVID-19 pandemic. For the first hypothesis, the proportion of Chinese respondents who used face masks was significantly higher than Poles. For the second hypothesis, the proportion of Polish respondents who report physical symptoms resembling COVID-19 infection, recent medical consultation, recent COVID-19 testing and hospitalization were higher than Chinese and these could be predisposing factors for anxiety, depression, and stress in Poles. The Chinese respondents had significantly higher IES-R scores than Poles. It is important to note that the Cronbach’s alpha value of Chinese version of IES-R was higher than 0.90 and it might suggest redundancy of items (41, 42).

Prior to the COVID-19 pandemic, the two countries had similar age standardized prevalence of depression and anxiety (18). In 2017, the age-standardized prevalence of depression in China and Poland were 3.4% and 2.3%, respectively. The age-standardized prevalence of anxiety in China and Poland were 3 and 3.4% (18). From 2005–2008 to 2016–2018, Poland and China had similar levels of increase in happiness scores (Poland: 0.445; China: 0.426) (43). For the third hypothesis, the factors associated with adverse mental health were different for Polish and Chinese respondents. For Polish respondents, male gender, and high level of education were protective factors while unemployment, retirement, physical symptoms resembling COVID-19 infection, recent medical consultation or COVID-19 testing, and long daily duration of home confinement (20–24 h) were risk factors for PTSD symptoms, anxiety, depression, or stress. In contrast, for Chinese respondents, male gender, student status, high education level, and physical symptoms resembling COVID-19 infection were risk factors. Polish respondents reported significantly higher levels of anxiety, depression, and stress as compared to the Chinese respondents. This could be due to the number of COVID cases and death per 1 million population was higher in Poland as compared with China during the recruitment periods (Poland: 32 COVID cases/1 million people; 0.42 deaths/1 million people on 26 March 2020; China: 12 COVID cases/1 million people; 0.25 deaths/1 million people on 2 February 2020) and different precautionary measures.

When the differences in mental health findings between the two countries were taken into considerations, this would provide important information about the effects of different precautionary measures. Chinese respondents were significantly more likely to wear face masks. The infrequent use of face mask but more knowledge about COVID-19 from other countries could be the underlying reasons for significantly more physical symptoms and more frequent consultation with a doctor, COVID-19 testing and hospitalization reported by Polish respondents. The proper use of face masks could be due to good public health education as Chinese respondents were more satisfied with health information in this study. Zhai (14) provided four benefits of wearing face masks (14). Firstly, from the view of air quality and ventilation, COVID-19 transmission through respiratory droplets produced by an infected person should also be treated as airborne (44). Case series in China showed that surface contact with infected materials might not be the main route for COVID-19 spread (14). Secondly, face masks are more efficient in preventing virus spread from asymptomatic patients. Third, there is no evidence of false sense of security and improper use of face masks that outweigh its benefits. Fourth, in view of shortage of face masks, China actively enhances the production of facemasks rather than discouraging its use. This could have instilled confidence, showing fewer COVID-19 cases and reduced the risk of adverse mental health among the Chinese. In April, 2020, it was clear that countries or cities with higher proportion citizens that used face masks had lower COVID-19 cases and controlled the epidemic much earlier than countries or cities that discouraged the use of face masks (45). The rising in number of COVID-19 cases and deaths in some countries have worsened the mental health of their citizens (46). The world opinion shifted in favour of face masks including the U.S. (47). During the recruitment period, the China government made it mandatory to wear face masks in public areas along with other precautionary measures throughout all regions in China. After the completion of recruitment of this study in Poland, the Polish government made it mandatory to wear face masks in public places on April 16 2020 (48). On May 30 2020, the Polish government decided to revoke the necessity of wearing face masks if the social distance of 2 m was preserved. This rule applied to both open and closed areas, various services (e.g., indoor restaurants and hair salons). Using face masks was still mandatory in public transport, stores, theaters, and churches (49). Since August 8 2020, due to the growing number of COVID-19 cases, Poland was divided into three zones with different level of restrictions and requirement to wear face masks in public spaces and the prohibition of organizing mass events [the red zones, with 9 counties of Silesia, Greater Poland, Lesser Poland, and Łódź Voivodships, where the restrictions are strictest and urgent, the yellow zones, with 10 counties of Silesia, Lesser Poland, Greater Poland, Subcarpatian, Swietokrzyskie, Łódź Voivodships, with intermediate levels of restrictions, and the green zones with the lowest levels of restriction and no urgent change in precaution (50)]. Polish researchers advocated for general public education campaigns on the proper use of face masks (51). Nevertheless, Polish held ambivalent views toward face masks with due to cultural reasons. It is generally difficult for Poles to accept the need to use them. This also applies to anti-smog and dust masks used to protect people employed in mining and industry. Before the outbreak of the COVID-19 pandemic, wearing protective masks was not a constant Polish practice as for the residents of many Chinese cities, whose awareness of the importance of wearing masks and the responsibility associated with the need to protect their own health and health of others are higher. The Chinese are more collectivistic than Poles, attached to social conformity and collective order (52). Therefore, it is easier for Chinese to accept wearing masks. From the social perspective, Poles perceive wearing masks as a sign of sickness and vulnerability. From the anthropological perspective, Poles believe that face mask is designed to make a person unreal and hide the identity (53). Our findings suggest that there is a need of health education for the adoption of the precautionary measures or promotion of the importance of the precautionary measures.

Besides precautionary measures, other risk and protective factors warrant further discussion. For Poles, female gender was associated with poor mental health and previous Polish study also found women were at higher risk of developing anxiety and depression (54). Nevertheless, previous study found that Polish men were less likely to consult general physicians about their psychiatric problems due to masculine issues (55). For Chinese respondents, male gender was associated with higher levels of anxiety, depression and stress. This finding corresponds to a recent study that found that higher percentage of Chinese males endured different degrees of depression (56). The deterioration of Chinese males’ depression during COVID-19 might be partly attributed to their negative attitude toward emotional openness and reluctance to consult mental health professionals (56), although mental health services were disrupted (27).

Unemployed Poles, especially those who had not earned minimum national wage before the COVID-19 pandemic and retired Poles worried that they would receive less allowance due to tight budget of the government. From cultural viewpoints, Poles enjoyed freedom and independence (57) and a long duration of home confinement affected their mental health. As Polish respondents were less satisfied with health information, more educated Poles were capable to research and analyze different sources of health and led to better mental health. In contrast, Poles with lower education were more likely to break precautionary measures, facing high risk of COVID-19 infection and adverse mental health (58). In contrast, Chinese respondents were satisfied with health information in general. COVID-19 could have more impact on Chinese students as compared to Polish students due to differences in the education system. Every year, millions of Chinese students have prepared for years for the national university entrance exam (*gaokao*) that have a significant impact on their future studies and career. Rarely, this high-stake exam was postponed due to COVID-19 (59), and the disruption in academic plans led to higher levels of anxiety and stress among Chinese students.

### Limitations

One major limitation is the cross-sectional nature of this study and it would be very interesting if researchers could follow-up Polish citizens since the Polish government made mandatory the use of face masks. Although we found Polish respondents demonstrated significantly higher DASS-21 subscale scores during the COVID-19 pandemic, we did not record demographic data regarding preexisting mental illness of the respondents. Poles could suffer from higher levels of psychiatric morbidity before the COVID-19 pandemic. The worry, stress, and anxiety in Polish respondents might exist prior to this study because of the information provided through the media especially since the pandemic had affected other European countries. Poles faced higher levels of social stigmatization of mental illness (60, 61) and underfunding of hospital treatment of depression (55). There is a potential risk of sampling bias because we could not reach out to potential respondents without Internet access in both countries. The respondent sampling method also compromised the representativeness of samples. Respondent Sampling Technique depends on existing study participants recruiting new participants from their acquaintances (snowballing). By virtue of the non-probabilistic nature of this technique, the sample is not representative of the underlying population. Both samples in China and in Poland were recruited in the same way, but distribution of demographic variables in both samples was different. Yet, these demographic differences between both samples could in part compensated through the use of stratified analysis and adjustment for confounders. The study population had different sociodemographic characteristics as compared to the general population. For Chinese, the male to female gender ratio in 2018 was 1.04:1 (62) but 67.3% of Chinese respondents were female. The proportion of study population with university education was higher than the general population. In addition, 72.9% of Chinese respondents in this study had university education while 17% of the Chinese general population had university education (63). Similarly, 87.9% of Polish respondents in this study had university education while 27% of the Polish general population had university education (63). We need to consider the above socio-demographic differences when interpreting the results of this study. Another limitation is that self-reported levels of psychological impact, anxiety, depression, and stress may not always be aligned with objective assessment by mental health professionals. Nevertheless, psychological impact, anxiety, depression, and stress are based on personal feelings, and self-reporting was paramount during the COVID-19 pandemic. Lastly, we could not calculate the response rate. For potential respondents who were not keen to participate the online survey, they would not provide any response and we cannot collect any information from them.

### Conclusion

During the COVID-19 pandemic, this cross-country study found that the proportion of Polish respondents who used masks were significantly less than Chinese. The infrequent use of face mask could be the contributing cause for significantly more physical symptoms and more frequent medical consultation, COVID-19 testing, and hospitalization reported by Polish respondents. Poles had significantly higher levels of anxiety, depression, and stress as compared to Chinese. Besides precautionary measures, unemployment, retirement, physical symptoms resembling COVID-19 infection, recent medical consultation or COVID-19 testing and long daily duration of home confinement were risk factors for PTSD symptoms, anxiety, depression, or stress for Polish respondents. There is a need of health education with scientific information from Polish health authority on the proper use of a face masks and reduce social stigma. However, this study was limited by the respondent sampling method that has compromised the representativeness of samples and the demographic differences between both samples.

## Data Availability Statement

The raw data supporting the conclusions of this article will be made available by the authors, without undue reservation.

## Ethics Statement

The studies involving human participants were reviewed and approved by Institutional Review Boards of SWPS University (Poland) and Huaibei Normal University (China). Written informed consent to participate in this study was provided.

## Author Contributions

CW and AC-C contributed equally to the paper and are co-first authors. CW, AC-C, DG, KA, MH, RP, and RH led the conception and design of the survey. CW, RP, and LX supported the training and supervision of data collection teams in China. AC-C, DG, KA, and MH supported the training and supervision of data collection teams in Poland. CW and RP led the data analysis with support from XW, YT, and JQ. LX conducted separate analysis of all data to ensure consistency. CW, AC-C, DG, RP, CH, RM, and RH conducted careful reviews of all analysis. AO-G, AC-C, DG, KA, and MH provided further information on cultural perspectives. CW, AC-C, RP, DG, KA, MH, RM, CH, and RH performed critical review of the manuscript. All authors contributed to the article and approved the submitted version.

## Funding

This study has the following funding sources: Huaibei Normal University, China, Ministry of Science and Higher Education in Poland under the 2019–2022 program ”Regional Initiative of Excellence”, project number 012/RID/2018/19 and National University of Singapore iHeathtech Other Operating Expenses (R-722-000-004-731).

## Conflict of Interest

The authors declare that the research was conducted in the absence of any commercial or financial relationships that could be construed as a potential conflict of interest.
